# A Study on the Mechanism of Milkvetch Root in the Treatment of Diabetic Nephropathy Based on Network Pharmacology

**DOI:** 10.1155/2020/6754761

**Published:** 2020-10-31

**Authors:** Chunli Piao, Qi Zhang, De Jin, Li Wang, Cheng Tang, Naiwen Zhang, Fengmei Lian, Xiaolin Tong

**Affiliations:** ^1^Shenzhen Hospital, Guangzhou University of Chinese Medicine (Futian), Shenzhen 518000, Guangdong, China; ^2^Changchun University of Chinese Medicine, Changchun 130000, Jilin, China; ^3^Guang'anmen Hospital, China Academy of Chinese Medical Science, Beijing 100000, China

## Abstract

Diabetic nephropathy (DN) is one of the most common complications of diabetes mellitus. Owing to its complicated pathogenesis, no satisfactory treatment strategies for DN are available. Milkvetch Root is a common traditional Chinese medicine (TCM) and has been extensively used to treat DN in clinical practice in China for many years. However, due to the complexity of botanical ingredients, the exact pharmacological mechanism of Milkvetch Root in treating DN has not been completely elucidated. The aim of this study was to explore the active components and potential mechanism of Milkvetch Root by using a systems pharmacology approach. First, the components and targets of Milkvetch Root were analyzed by using the Traditional Chinese Medicine Systems Pharmacology database. We found the common targets of Milkvetch Root and DN constructed a protein-protein interaction (PPI) network using STRING and screened the key targets via topological analysis. Enrichment of Gene Ontology (GO) pathways and Kyoto Encyclopedia of Genes and Genomes (KEGG) pathways were analyzed. Subsequently, major hubs were identified and imported to the Database for Annotation, Visualization and Integrated Discovery for pathway enrichment analysis. The binding activity and targets of the active components of Milkvetch Root were verified by using the molecular docking software SYBYL. Finally, we found 20 active components in Milkvetch Root. Moreover, the enrichment analysis of GO and KEGG pathways suggested that AGE-RAGE signaling pathway, HIF-1 signaling pathway, PI3K-Akt signaling pathway, and TNF signaling pathway might be the key pathways for the treatment of DN; more importantly, 10 putative targets of Milkvetch Root (AKT1, VEGFA, IL-6, PPARG, CCL2, NOS3, SERPINE1, CRP, ICAM1, and SLC2A) were identified to be of great significance in regulating these biological processes and pathways. This study provides an important scientific basis for further elucidating the mechanism of Milkvetch Root in treating DN.

## 1. Introduction

According to an epidemiological survey, the number of Chinese adults of ages over 18 who suffered from diabetes mellitus (DM) was 10.4% in 2013, ranking the first in the world [1]. Diabetic nephropathy (DN) is one of the most common complications of DM [2]. The incidence rate of DN is increasing rapidly along with that of DM. DN is the most common cause of end-stage renal disease (ESRD) in many parts of the world, including Europe, Japan, and the United States, wherein diabetic patients accounted for 25% to 45% of all patients enrolled in end-stage renal disease programs [3]. Glomerular damage and proteinuria that are associated with DM cause tubulointerstitial damage, which eventually lead to ESRD [4, 5]. The early onset of DN is insidious and difficult to detect early. Moreover, once DN has reached the end of its clinical development, it is difficult to reverse. At present, the treatment of diabetic nephropathy mainly includes strict control of blood glucose, blood pressure, and antidiabetic drugs, which all can only delay the progress of renal damage, as there are no new therapies that can directly treat DN [6]. Moreover, studies have shown that inhibitors of the Renin–Angiotensin–Aldosterone System (RAAS) have significant side effects, including hyperkalemia; these effects limit the use of inhibitors in a significant proportion of patients with DN [7]. Studies have also shown that ACEIs (angiotensin-converting enzyme inhibitor) and ARBs (angiotensin receptor blocker) have many side effects such as acute renal injury and angioedema [8]. Therefore, more effective and safer therapeutic strategies for treating DN are required.

Traditional Chinese medicine (TCM) has been used to treat various diseases for thousands of years. TCM also has unique advantages in diabetes and is widely used in clinical practice in China [9, 10]. Milkvetch Root (Huang Qi in Chinese), also known as Radix Astragali, is a TCM from Mongolian Milkvetch or Membranous Milkvetch. Milkvetch Root has been reported to replenish qi, consolidate superficial resistance, induce diuresis, toxin elimination, discharge pus, relieve soreness, and increase muscle mass [11]. In TCM, it is often used as antiperspirant, diuretic, and supplement in treating various diseases such as abscess, nephritis, diabetes, hypertension, cirrhosis, leukemia, and uterine cancer [12]. In recent years, the therapeutic effect of Milkvetch Root on DN has attracted attention. A clinical study indicated that the adjunctive use of Milkvetch Root might be effective and tolerated for the short-term reduction of albuminuria, proteinuria, and serum creatinine in DN patients [13]. A basic study suggested that Astragaloside IV ameliorates high glucose-mediated renal tubular epithelial-mesenchymal transition by blocking the mTORC1/p70S6K signaling pathway in HK-2 cells [14]. Other studies showed that Astragaloside IV ameliorated albuminuria, mesangial cell proliferation, basement membrane thickening, and podocyte foot process effacement in iatrogenic hyperinsulinemia rats [15]. These studies provide a scientific basis for the clinical application of Milkvetch Root in treating DN; however, the molecular mechanism of Milkvetch remains unclear. Therefore, the active ingredients and molecular mechanisms of Milkvetch Root for the treatment of DN must be elucidated.

Network pharmacology, which is based on the interaction network of diseases, genes, target proteins, and drugs, is a systematic analytical method [16]. In recent years, network pharmacology has been used widely in TCM research [17]. For example, the network pharmacology approach was used to define the active components and potential targets in Mulberry leaf for the treatment of diabetes [18]. It can reveal the action mechanism of a drug through the combination of computational biology, systems biology, and “omics” technologies [19]. It also has transformed the concept of drug discovery from “one target, one drug” to “network target, multicomponent therapy.”

**Figure 1:**
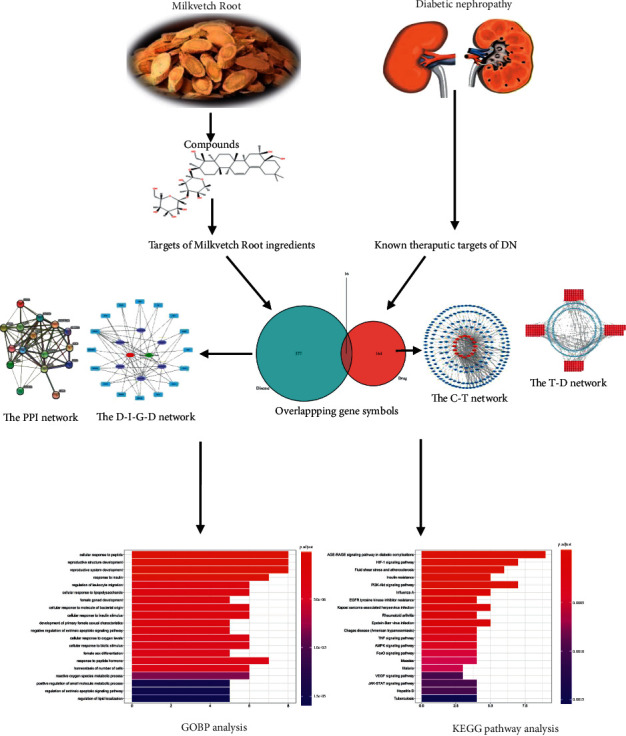
Flowchart of a network pharmacology-based strategy to investigate the pharmacologic mechanism of Milkvetch Root for treatment of diabetic nephropathy.

In summary, we used network pharmacology to analyze the active ingredients, drug targets, and key pathways of Milkvetch Root to treat DN. This study aimed to further elucidate the mechanism of Milkvetch Root in treating DN and present new ideas and theoretical basis. The workflow of the network pharmacology approach used in the present study is illustrated in Figure 1.

## 2. Materials and Methods

### 2.1. Data Preparation

All components related to Milkvetch Root were screened by using Traditional Chinese Medicine Systems Pharmacology Database and Analysis Platform (TCMSP, http://lsp.nwu.edu.cn/tcmsp.php) [20]. Five important pharmacology-related properties, including oral bioavailability (OB), intestinal epithelial permeability (Caco-2 cells), drug-likeness (DL), blood-brain barrier (BBB), drug half-life (HL), and Lipinski's rule (LR), were considered for the screening and evaluation of compounds in using TCSMP. The TCMSP database contains 500 kinds of Chinese herbal medicines, and 30069 ingredients are registered in Chinese Pharmacopoeia (2010 edition). Moreover, 87 herbal ingredients of Milkvetch Root were identified in this process.

### 2.2. Screening of Active Ingredients

The key parameters used for screening in the database were oral bioavailability (OB) and drug-likeness (DL), and the active components of Milkvetch Root were screened. OB is an important property for the objective evaluation of the internal quality of drugs. When an ingredient has a high OB, the likelihood that it can be used clinically is also high [21]. Molecules with OB ≥ 30% were considered to have good OB in the present study. Drug-likeness (DL) is a qualitative concept used in drug design and helps optimize pharmacokinetics and drug properties such as solubility and chemical stability. A database-dependent DL evaluation approach based on Tanimoto coefficient was applied and shown as *T*(*a*, *b*)=(*a*, *b*)/(|*a*|^2^+|*b*|^2^ − *a* × *b*). In this equation, *a* represents the molecular descriptors of herbal compounds and *b* represents the average molecular properties of all compounds in DrugBank. Components with DL ≥ 0.18 were selected. In this study, the compounds of Milkvetch Root that had OB ≥ 30% and DL ≥ 0.18 were considered as active components.

### 2.3. Targets of Active Ingredients of Milkvetch Root

The targets of the active components of Milkvetch Root were queried against the TCMSP database. We removed redundant information, and the targets were transformed using the UniProt knowledge database [22] (UniProt, https://www.uniprot.org/) with *Homo sapiens* as the selected species. At the end, we can get the right genetic symbols.

### 2.4. Identification of Gene Targets for DN

We collected the gene targets of DN from four sources. The first source was the GeneCards v4.14 [23] (http://www.genecards.org/, 2020.03.20). A correlation score of ≥30 was used as the screening parameters, and the returned items from the screening were used as the candidate target genes of the disease. The rest of the sources were DrugBank v4.3 [24] (http://www.drugbank.ca/, 2020.03.20), Online Mendelian Inheritance in Man (OMIM) [25] (http://www.omim.org/, 2020.03.20), and PharmGkb (https://www.pharmgkb.org/, 2020.03.20) [26]. The keyword “diabetic nephropathy” or “DN” was input to obtain the gene names related to diabetic nephropathy. We removed the duplicates of search elements in these four databases.

### 2.5. Network Construction

We intersected the returned drug targets with the genes that were associated with DN and illustrated the intersection using a Venn diagram. Subsequently, we built a compound-target network by linking candidate compounds to their corresponding targets. Moreover, we built a target-disease network by linking diseases to candidate targets that are associated with them. Furthermore, we built a drug-ingredient-gene-disease (DIGD) network based on the interactions among drugs (Milkvetch Root), ingredients, gene symbols, and disease (DN).

We selected three parameters to evaluate the topological features of every node in the interaction network: degree reflects the number of connections between network nodes and other nodes; betweenness is the ratio of the number of shortest paths through a point to the total number of shortest paths in the network; and closeness is the distance between a node and another node. Degree, betweenness, and closeness are the main topological parameters used to measure the importance of a node in a network and determine whether a target protein is an important basis for key targets [27]. Therefore, key targets of C-T network and T-D network were analyzed topology parameter characterization with Network Analyzer. The degree, betweenness, and closeness value were set to the median degree. Finally, the networks were constructed using Cytoscape v3.7.2 (http://www.cytoscape.org/) [28].

### 2.6. Construction of a Protein-Protein Interaction (PPI) Network

To determine the interactions between target proteins, the target genes of the relevant components in the Milkvetch Root were queried against the STRING database (http://string-db.org, v11) [29] to obtain information on PPI. Gene symbols were returned from the query using the “multiple proteins” option and *Homo sapiens* as the organism option. We selected medium-confidence data of >0.4. The returned protein interaction data were analyzed in Cytoscape 3.7.2 to build a PPI network.

### 2.7. Enrichment of Gene Ontology (GO) Pathway and the Kyoto Encyclopedia of Genes and Genomes (KEGG) Pathway

To further identify the related effects of Milkvetch Root in treating diabetic nephropathy, we used GO biological process enrichment analysis to evaluate the targets of Milkvetch Roots and KEGG metabolic pathway enrichment analysis to determine the main metabolic pathway of Astragalus in treating DN. We performed further analyses using R software v3.6.2 (http://bioconductor.org/) [30] and its cluster profiler package.

### 2.8. Molecular Docking

Molecular docking is a process through which small molecules are docked into the macromolecular structures for scoring its complementary values at the binding sites [31]. High-resolution crystal structures of the active components and their corresponding bioactive ligands were downloaded from the Protein Data Bank (PDB) [32]. SYBYL software is simulation software for the molecule docking analysis of small molecules and biological macromolecules [33]. We used SYBYL software to evaluate the potential binding between DN targets and Milkvetch Root compounds. We used SYBYL software and optimized the mechanisms of small molecule compounds, hydrogenation, charging, extraction of ligand small molecules, repair of side chain, and hydrogenation. Then, we used the Surflex-Dock module molecular docking [34]. During the docking, the threshold parameter was set at 0.5 and other parameters were set at default values. The results of molecular docking were evaluated according to a total score; the total score was expressed in −log10 (Kd) units, and the value of total score equal to 5 was taken as the threshold value.

## 3. Results

### 3.1. Screening of Active Ingredients

The active components of Milkvetch Root were retrieved from the TCMSP database, and 87 related components were obtained. 20 related components were identified to have OB ≥ 30% and DL ≥ 0.18. Altogether, 20 components were considered as the active ingredients of Milkvetch Root (Table 1).

### 3.2. Target Prediction and Analysis

**Figure 2:**
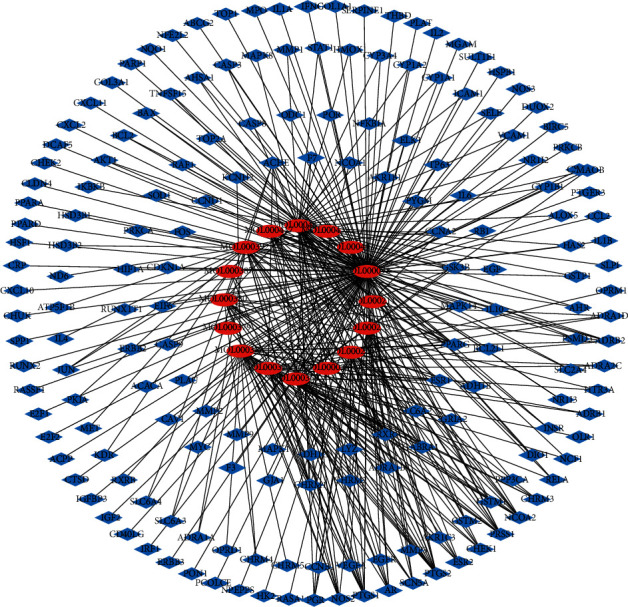
The C-T network that consists of 16 nodes and 360 targets. Red and blue nodes denote the compounds and targets, respectively.

**Figure 3:**
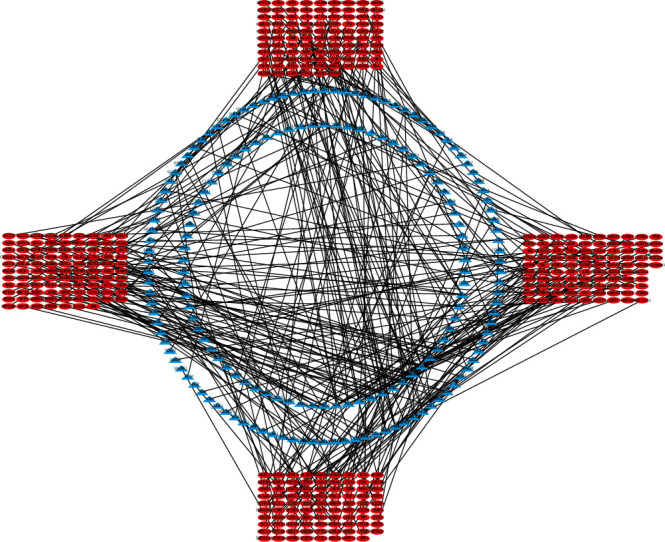
The T-D network that consists of 393 nodes and 360 targets. Red and blue nodes denote the diseases and targets, respectively.

**Figure 4:**
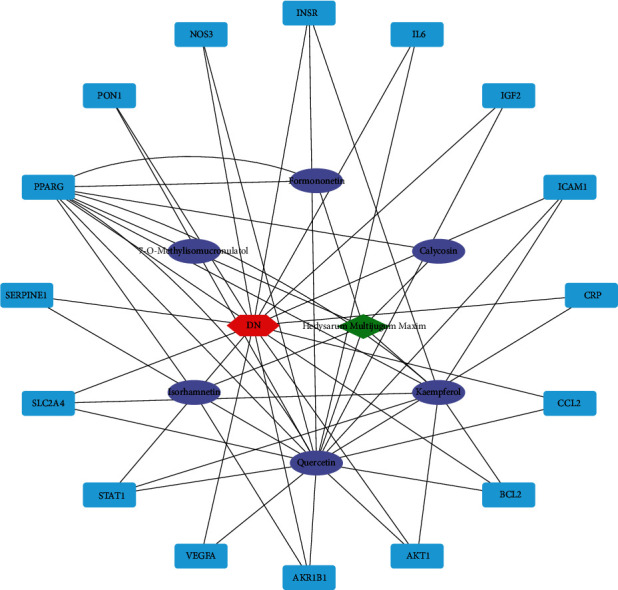
The DIGD network. The green node represents Milkvetch Root and the red node represents DN. The 6 violet nodes represent the active ingredients in Milkvetch Root. The 16 blue nodes represent the overlapping gene symbols between the disease and drug. The edges denote that nodes can interact with each other.

**Figure 5:**
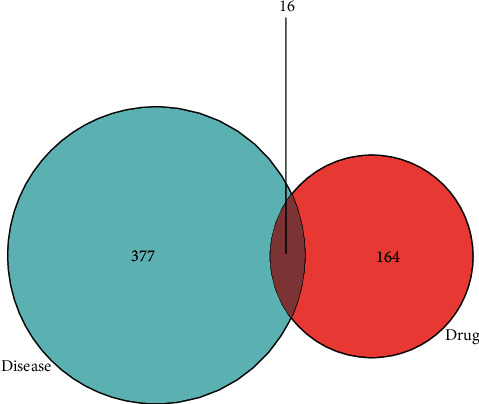
The 16 matching targets of the related targets in Milkvetch Root on DN.

Twenty active components were obtained from Milkvetch Root, and 393 potential targets of these components were identified. All the targets related to DN were queried against four databases: GeneCards, DrugBank, OMIM, and PharmGkb. We used Cytoscape 3.7.2 software to build a compound-target (C-T) network using the active ingredients of Milkvetch Root and their targets. Concurrently, we also used Cytoscape 3.7.2 software to analyze the relationship between the targets of Milkvetch Root and DN and constructed a target-disease (T-D) network (Figures 2 and 3). In the C-T network, the median values of “degree,” “betweenness,” and “closeness” were 1,0, 0.39, and in the T-D network, the median values of “degree,” “betweenness,” and “closeness” were 1,0, 0.667, respectively. The final results are shown in Tables 2 and 3. To further study the mechanism of Milkvetch Root in treating diabetic nephropathy, we also constructed a DIGD network using Cytoscape 3.7.2 software (Figure 4). The green node represents Milkvetch Root, and the red node represents DN. Moreover, the 6 violet nodes represent the active ingredients of Milkvetch Root; the 16 blue nodes represent the overlapping gene symbols between the disease and drug. The edges denote that the nodes can interact with each other. The network shows that the drug may indirectly regulate disease-related proteins while Milkvetch Root can directly affect these proteins. There were 16 overlaps among 393 disease gene symbols and 180 drug gene symbols (Figure 5). In other words, these 16 genetic symbols may be the key targets of Milkvetch Roots in treating DN.

### 3.3. Analyses of a PPI Network

**Figure 6:**
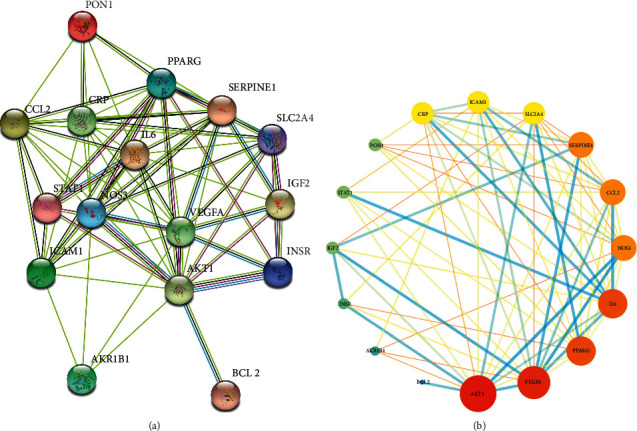
(a) PPI network of 16 nodes and 71 edges established in the STRING database. (b) PPI network of 16 nodes and 71 edges established in Cytoscape 3.7.2.

We constructed a PPI network consisting of 16 nodes and 71 edges (Figure 6(a)). In this network, nodes represent target proteins, and each edge represents a protein-protein interaction. The average node degree in this PPI network is 8.88; the degree of each node represents the number of targets that are connected to the target. Shown in Figure 6(b) is a PPI network constructed using Cytoscape 3.7.2. In this network, node sizes and colors reflect the number of combined targets (degree).

**Figure 7:**
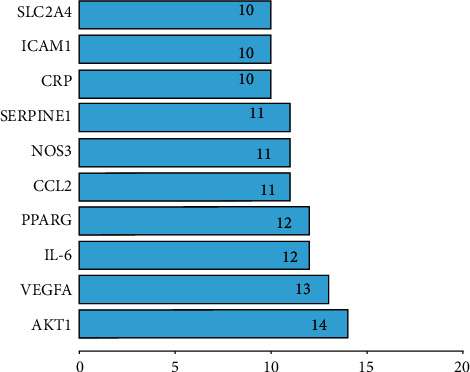
The bar plot of the PPI network. The *x*-axis represents the number of neighboring proteins of the target protein. The *y*-axis represents the target protein.

We took the first 10 proteins in the PPI network, which includes RAC-alpha serine/threonine-protein kinase (AKT1), vascular endothelial growth factor A (VEGFA), interleukin-6 (IL-6), peroxisome proliferator-activated receptor gamma (PPARG), C-C motif chemokine 2 (CCL2), nitric oxide synthase, endothelial (NOS3), plasminogen activator inhibitor 1 (SERPINE1), C-reactive protein (CRP), intercellular adhesion molecule 1 (ICAM1) and solute carrier family 2, and facilitated glucose transporter member 4 (SLC2A4) (Figure 7). As shown in Figure 7, AKT1 may be related to 14 other proteins, VEGFA may be related to 13 other proteins, and IL-6 and PPARG may be related to the 12 other proteins. CCL2, NOS3, and SERPINE1 may be related to the 11 other proteins. CRP, ICAM1, and SLC2A4 may be related to 10 other proteins. These ten proteins are the focus of our research on PPIs.

### 3.4. Analyses of Enrichment of GO Pathways

**Figure 8:**
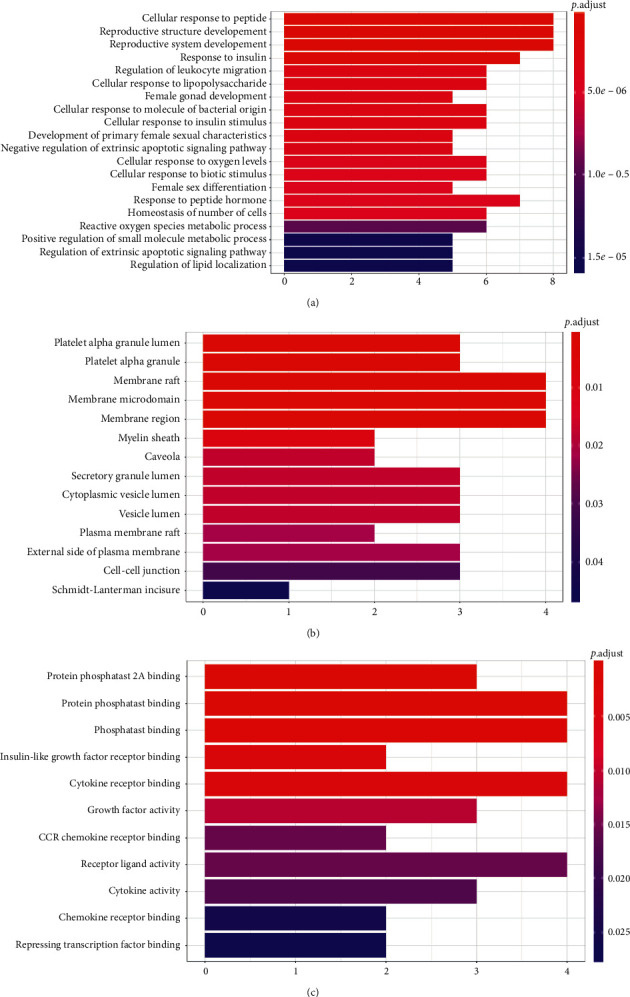
(a) Main 20 GO biological process. (b) Main 14 GO cellular component. (c) Main 11 GO molecular function. *p* < 0.05.

GO analysis is a useful bioinformatics tool for characterizing molecular function (MF), cellular components (CC), and biological process (BP) of genes. Analyses of the enrichment of the GO pathway were carried out using R software (Figures 8(a)–8(c)) (*p* < 0.05). In the graph, the vertical axis represents the GO term. The horizontal axis represents the number of genes in the term. The increasing intensity of the red color indicates a decreasing *p*. Adjust value indicates a higher significance. The 16 overlapping gene symbols were mapped to 977 pathways after the enrichment of the GOBP pathway. We identified the first 20 terms from small to large according to *p* values. The results indicate that numerous biologic processes were involved in DN treatment, including cellular response to the peptide (GO:1901653), reproductive structure development (GO:0048608), reproductive system development (GO:0061458), response to insulin (GO:0032868), regulation of leukocyte migration (GO:0002685), cellular response to lipopolysaccharide (GO:0071222), female gonad development (GO:0008585), cellular response to molecule of bacterial origin (GO:0071219), cellular response to insulin stimulus (GO:0032869), and development of primary female sexual characteristics (GO:0046545). Moreover, 14 pathways were enriched in the GOCC pathway, including extracellular space, cytosol, and extracellular area. 11 pathways were enriched in the GOMF pathway, including enzyme binding, protein binding, and similar protein binding.

### 3.5. Analyses of Enrichment of the KEGG Pathway

**Figure 9:**
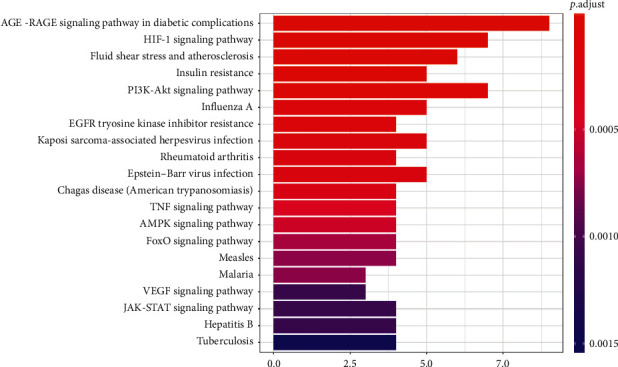
KEGG pathway enrichment analyses. The *x*-axis represents the counts of the target symbols in each pathway. The *y*-axis represents the main pathway (*p* < 0.05).

Analyses of the enrichment of the KEGG pathway were performed using R software (Figure 9) (*p* < 0.05). In the graph, the vertical axis represents the KEGG pathway. The horizontal axis represents the number of genes in the term. We identified the first 20 terms from small to large according to *p* values. The first 10 items are as follows: AGE-RAGE signaling pathway in diabetic complications (hsa04933), HIF-1 signaling pathway (hsa04066), fluid shear stress and atherosclerosis (hsa05418), insulin resistance (hsa04931), PI3K-Akt signaling pathway (hsa04151), influenza A (hsa05164), EGFR tyrosine kinase inhibitor resistance (hsa01521), Kaposi sarcoma-associated herpesvirus infection (hsa05167), rheumatoid arthritis (hsa05323), and Epstein–Barr virus infection (hsa05169).

### 3.6. Molecular Docking Verification

**Figure 10:**
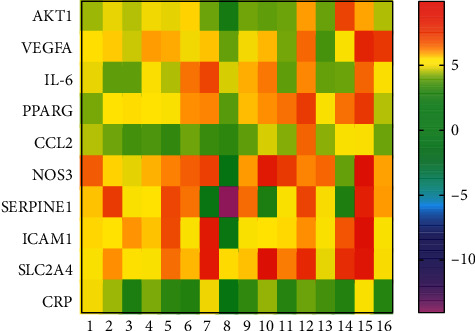
The cluster heat map of molecular docking between compounds and hub genes. One to 16 represents quercetin, kaempferol, formononetin, isorhamnetin, calycosin, 7-O-methylisomucronulatol, (3S,8S,9S,10R,13R,14S,17R)-10,13-dimethyl-17-[(2R,5S)-5-propan-2-yloctan-2-yl]-2,3,4,7,8,9,11,12,14,15,16,17-dodecahydro-1H-cyclopenta[a]phenanthren-3-ol, mairin, jaranol, hederagenin, 3,9-di-O-methylnissolin, 9,10-dimethoxypterocarpan-3-O-*β*-D-glucoside, (6aR,11aR)-9,10-dimethoxy-6a, 11a-dihydro-6H-benzofurano [3,2-c]chromen-3-ol, bifendate, FA, and 1,7-dihydroxy-3,9-dimethoxy pterocarpene, respectively.

In this docking assay, ten human receptors were retrieved from PDB: AKT1 (PDB ID: 1UNQ: 0.98 Å), VEGFA (PDB ID: 3V2A: 3.20 Å), IL-6 (PDB ID: 4CNI: 2.20 Å), PPARG (PDB ID: 3E00 : 3.10 Å), CCL2 (PDB ID: 1DOM), NOS3 (PDB ID: 1NIW: 2.05 Å), SERPINE1 (PDB ID: 4AQH: 2.40 Å), CRP (PDB ID: 1GNH: 3.00 Å), ICAM1 (PDB ID: 5MZA: 2.78 Å), and SLC2A4 (PDB ID: 5EQG: 2.90 Å). 10 hub genes were inputted into SYBYL 2.1 for molecular docking verification. The results of the molecular docking are shown in Table 4. The docking scores were larger than 5, which showed that they possessed good binding activity. Furthermore, the results are presented in the form of a cluster heat map (Figure 10).

## 4. Discussion

Network pharmacology is a rapidly emerging discipline. It has also transformed the concept of drug discovery from “one target, one drug” to “network target, multicomponent therapy” [35]. Because of the advantages of network pharmacology research strategy, new and innovative ways for the development of traditional Chinese medicine opened. The aim of this study was to analyze the active components, targets, and related signaling pathways of Milkvetch Root in improving glycolipid metabolism of diabetic nephropathy by using systems pharmacology and to explore the possible mechanism of action of the components of Milkvetch Roots.

Using network pharmacological analysis, we identified 20 active components in Milkvetch Root and predicted 180 potential targets. The results of the T-D network analysis showed 360 edges in the network, which represent the interaction between the active components and targets. Among the components, quercetin had the greatest number of potential targets with 136, followed by kaempferol with 51 potential targets. Other components such as 7-O-methylisomucronulatol, formononetin, isorhamnetin, and (6aR, 11aR)-9, 10-dimethoxy-6a, 11a-dihydro-6H-benzofurano [3, 2-c] chromen-3-ol had 33, 28, 25, and 19 corresponding targets, respectively. The corresponding targets of the active ingredients include AKT1, VEGFA, IL-6, PPARG, and NOS3. Moreover, molecular docking showed that the binding strength of 16 active components of Milkvetch Root to their target proteins was as follows: PPARG > VEGFA > IL-6 > AKT1> NOS3. We infer that quercetin has good interactions with NOS3, VEGFA, and SERPINE1. Kaempferol has good interactions with PPARG and VEGFA. 7-O-Methylisomucronulatol has good interactions with IL-6, NOS3, and SERPINE1.

Quercetin, a flavonoid, is a potent antioxidant found in common medicinal herbs and possesses a wide spectrum of biologic activities [36]. It also has antioxidant, hypoglycemic, hypolipidemic, tumor suppression, and anti-inflammatory effects [37, 38]. One study showed that quercetin liposome or free quercetin could prevent weight loss, decrease kidney hypertrophy index, decrease blood glucose level, and decrease 24-hour urine protein levels in diabetic nephropathy model rats [39]. Kaempferol is a natural peroxisome proliferator-activated receptor-*γ* (PPAR*γ*) agonist, and PPAR*γ* agonists have become common drugs in the treatment of diabetes and its complications [40]. Kaempferol has a similar hypoglycemic effect to rosiglitazone; however, its adverse reactions are significantly lower than those of the latter. It can improve the glucose uptake of 3T3-L1 cells, control blood glucose, and ameliorate the damage from oxidative stress in the kidney caused by glucose metabolism disorder [41]. Studies have also suggested that kaempferol can work as a RhoA/Rho kinase inhibitor and may attenuate the progression of diabetic complications with emphasis on DN [42]. Formononetin, a polyphenolic compound, is a molecule that increases the expression of SIRT1 in kidney tissues of diabetic patients and an effective molecule for controlling nephropathy in type 2 diabetes mellitus [43]. 7-O-Methylisomucronulatol has a similar pharmacological effect to formononetin; it can prevent and treat DN by inhibiting the proliferation of mesangial cells and the production of nitric oxide [44].

Isorhamnetin can inhibit the NF-*κ*B signaling activity, decrease the production of inflammatory mediators, and attenuate oxidative stress in diabetic rats and glomerular mesangial cells (GMCs), thus reducing urinary albumin filtration and renal damage and improving renal pathological changes among other effects [45]. This shows the complex network relationship between drugs and targets and verifies that Milkvetch Root plays a role in improving DN in a multicomponent and multitarget way.

In addition, quercetin, kaempferol, formononetin, and isorhamnetin are all flavonoids. Studies on the mechanism of action have suggested that flavonoids can improve the metabolism of sugar and lipid, enhance insulin resistance, inhibit the activity of relevant glucose metabolic enzymes, and escape oxidative damage of DM [46, 47]. We emphasize that these components may be the main components of Milkvetch Root. The flavonoids of TCM may be novel components for the treatment of diabetic nephropathy and has broad prospects for development.

The analysis of protein interaction showed that there was a correlation between AKT1, VEGFA, IL-6, PPARG, and NOS3. First of all, there are three isoforms of AKT: AKT1 (PKB*α*), AKT2 (PKB*β*), and AKT3 (PKB*γ*), which each have their own physiologic functions [48]. The protein kinase AkT, also known as protein kinase B (PKB), has been shown to regulate a variety of cell functions and is particularly important for glucose metabolism, cell growth, and cell survival. Therefore, changes in its expression or activity are thought to be involved in the pathogenesis of diabetes and DN [49].

In humans, there are five secreted glycoproteins that make up the VEGF family member: VEGFA, VEGF-B, VEGF-C, VEGF-D, and placental growth factor (PlGF) [50]. Previous studies have demonstrated that angiotensin type 1 receptor blocker (ARB) can inhibit the synthesis of VEGF mediated by Ang-II and can effectively treat diabetic nephropathy [51]. VEGFA is an important regulator of angiogenesis and vascular permeability with a possible pathogenic role in diabetic nephropathy [52]. VEGFA is essential for the normal growth of podocytes. When the expression of VEGFA was lower than the normal level, the podocytes were damaged [53]. In conclusion, the blockade of VEGFA can effectively restore renal function in diabetic nephropathy.

IL-6, in the pathogenesis of DN, is associated with insulin resistance. A study has suggested that IL-6 affects the dynamics of the extracellular matrix and may increase the glomerular basement membrane and endothelial permeability [54]. Current evidence suggests that IL-6 responses are mediated via gp130-STAT3 dependent mechanisms, which, on one hand, trigger the transition from innate to adaptive immune response and on the other hand act locally for tissue remodeling and immune cell infiltration [55]. Therefore, the regulation of IL-6 target is of great significance in the treatment of DN.

PPARG is a transcription factor that is activated by ligands. Currently, it has three subtypes: PPAR*α*, PPAR*β*, and PPAR*γ* [56]. Some studies have found that PPARG is a risk of progression of diabetic nephropathy in China [57]. Nitric oxide (NO) has been closely linked to the kidney according to renal hemodynamics regulation, renin secretion, inhibition of renal tubular sodium reabsorption, renal tubular glomerular feedback (TGF), and renal sympathetic nerve activity [58, 59]. The synthesis of NO in vivo has been reported to be closely linked to nitric oxide synthetase (NOS2/3) [60]. The active components act on NOS related targets, enhance the biological activity of NOS, and restore the pathways that downregulate the expression of inflammatory factors, thereby reducing creatinine level, protein filtration rate, and protect the kidney [61].

In this study, molecular docking and network analyses showed that all protein-pathway pairs were distributed among oxidative stress, inflammation, metabolism, immune system, apoptosis, and multiple pathways. For instance, oxidative stress and inflammation prompted by hyperglycemia are key initiators that lead to renal damage and nephropathy [62, 63]. AGE-RAGE (diabetes), HIF-1, PI3K-Akt, and TNF signaling pathways are responsible for the therapeutic effects on DN. Some studies have confirmed that AGE-RAGE signaling pathway is a signaling mechanism in the pathogenesis of diabetes and its complications [64]. It can aggravate the vascular damage implicated by diabetes through oxidative stress [65] and increase the risk of renal function deterioration and cardiovascular events, thereby leading to an increase in mortality [66]. HIF may activate during the early stage of DN under hypoxia and stimulate the proliferation and aggregation of inflammatory factors in the damaged kidney; this paves way for renal fibroblast scarring [67, 68]. Moreover, HIF can be combined with fibrosis-promoting genes such as collagen 1, connective tissue growth factor, and plasminogen activator inhibitor 1 to generate interstitial collagen, reduce the degradation of the extracellular matrix (ECM), and eventually lead to renal fibrosis [69]. PI3K-Akt signaling pathway has been indicated as the source of glomerular hypertrophy and ECM accumulation [70]. PI3K can activate its downstream molecule Akt, which further phosphorylates fox OS, GSK-3, Bad, mTOR, and other proteins to cause a cascade reaction that plays a key role in the accumulation of extracellular matrix, mesangial cell proliferation, epithelial-mesenchymal transformation, and other aspects of diabetic nephropathy [71, 72]. TNF-*α* can stimulate the aggregation and adhesion of inflammatory cells, increase the permeability of small blood vessels, and damage the glomeruli through inflammatory reactions [73].

## 5. Conclusions

In this study, the mechanism of astragalus in treating DN was analyzed by using systems pharmacology approaches. We found six active ingredients that can directly affect diabetic nephropathy targets; we also found ten potential targets for the treatment of DN. We infer that the AGE-RAGE signaling pathway in diabetic complications, HIF-1 signaling pathway, PI3K-Akt signaling pathway, and TNF signaling pathway in diabetic complications serve as the key points and principal pathways for DN treatment. Altogether, we systematically explored how Milkvetch Root may affect DN treatment. We found that Milkvetch Root has multiple targets and approaches for treating DN. Such data provide the basis for multi-ingredient synergies in future research.

## Figures and Tables

**Table 1:** A total of 20 ingredients were selected as the details of the active ingredients of Milkvetch Root in this study.

Number	Mol ID	CAS number	Components	OB(%)	DL
1	MOL000211	472-15-1	Mairin	55.38	0.78
2	MOL000239	3301-49-3	Jaranol	50.83	0.29
3	MOL000296	465-99-6 474-58-8	Hederagenin	36.91	0.75
4	MOL000033	64997-52-0	(3S,8S,9S,10R,13R,14S,17R)-10,13-Dimethyl-17-[(2R,5S)-5-propan-2-yloctan-2-yl]-2,3,4,7,8,9,11,12,14,15,16,17-dodecahydro-1h-cyclopenta[a]phenanthren-3-ol	36.23	0.78
5	MOL000354	480-19-3	Isorhamnetin	49.6	0.31
6	MOL000371	15689655	3,9-di-O-Methylnissolin	53.74	0.48
7	MOL000374	N/A	5′-Hydroxyiso-muronulatol-2′,5′-di-O-glucoside	41.72	0.69
8	MOL000378	N/A	7-O-Methylisomucronulatol	74.69	0.3
9	MOL000379	94367-42-7	9,10-Dimethoxypterocarpan-3-O-*β*-D-glucoside	36.74	0.92
10	MOL000380	73340-41-7 94367-42-7	(6aR,11aR)-9,10-Dimethoxy-6a,11a-dihydro-6h-benzofurano[3,2-c]chromen-3-ol	64.26	0.42
11	MOL000387	73536-69-3	Bifendate	31.1	0.67
12	MOL000392	485-72-3	Formononetin	69.67	0.21
13	MOL000398	N/A	Isoflavanone	109.99	0.3
14	MOL000417	20575-57-9	Calycosin	47.75	0.24
15	MOL000422	520-18-3	Kaempferol	41.88	0.24
16	MOL000433	33609-88-0 59-30-3	FA	68.96	0.71
17	MOL000438	64474-51-7	(3R)-3-(2-Hydroxy-3,4-dimethoxyphenyl)chroman-7-ol	67.67	0.26
18	MOL000439	N/A	Isomucronulatol-7,2′-di-O-glucosiole	49.28	0.62
19	MOL000442	N/A	1,7-Dihydroxy-3,9-dimethoxy pterocarpene	39.05	0.48
20	MOL000098	73123-10-1 74893-81-5 117-39-5	Quercetin	46.43	0.28

**Table 2:** Key genes and compounds in the C-T network obtained by topological attribute analysis.

Name	Betweenness centrality	Closeness centrality	Degree
MOL000098	0.77966423	0.6372549	136
MOL000442	0.00006485	0.34210526	3
MOL000422	0.1540125	0.41313559	51
GSTM2	0.00145981	0.41313559	2
GSTM1	0.00145981	0.41313559	2
DIO1	0.00145981	0.41313559	2
INSR	0.00145981	0.41313559	2
NR1I3	0.00145981	0.41313559	2
SLC2A4	0.00145981	0.41313559	2
PSMD3	0.00145981	0.41313559	2
AHR	0.00145981	0.41313559	2
GSTP1	0.00145981	0.41313559	2
HAS2	0.00145981	0.41313559	2
ALOX5	0.00145981	0.41313559	2
CYP1B1	0.00145981	0.41313559	2
NR1I2	0.00145981	0.41313559	2
VCAM1	0.00145981	0.41313559	2
SELE	0.00145981	0.41313559	2
ICAM1	0.00145981	0.41313559	2
CYP1A1	0.00145981	0.41313559	2
CYP1A2	0.00145981	0.41313559	2
CYP3A4	0.00145981	0.41313559	2
HMOX1	0.00145981	0.41313559	2
STAT1	0.00145981	0.41313559	2
MMP1	0.00145981	0.41313559	2
CASP3	0.00145981	0.41313559	2
AHSA1	0.00145981	0.41313559	2
TNFSF15	0.00145981	0.41313559	2
BAX	0.00145981	0.41313559	2
BCL2	0.00145981	0.41313559	2
AKT1	0.00145981	0.41313559	2
JUN	0.00851752	0.44520548	3
KCNH2	0.00513961	0.42951542	2
ADRB2	0.0182466	0.45560748	6
NCF1	0.00273953	0.41139241	2
RELA	0.00486558	0.43526786	3
MAOB	0.00867608	0.43141593	3
ACHE	0.01869555	0.47560976	6
F7	0.00486558	0.43526786	3
AKR1B1	0.00273953	0.41139241	2
PPARG	0.02091558	0.47794118	9
RXRA	0.02752522	0.47794118	8
GABRA1	0.0242005	0.47794118	7
NCOA2	0.0331118	0.48507463	10
PRSS1	0.03297969	0.49242424	10
PTGS2	0.06147036	0.51315789	13
SCN5A	0.01714627	0.45990566	6
AR	0.02264676	0.48029557	7
PTGS1	0.05214304	0.5078125	11

**Table 3:** Key genes in the T-D network obtained by topological attribute analysis.

Name	Betweenness centrality	Closeness centrality	Degree
SERPINE1	1	1	2
NOS3	1	1	2
GSTM2	1	1	2
GSTM1	1	1	2
DIO1	1	1	2
NR1I3	1	1	2
SLC2A4	1	1	3
PSMD3	1	1	2
AHR	1	1	2
GSTP1	1	1	2
INSR	0.83333333	0.8	3
ALOX5	1	1	2
CYP1B1	1	1	2
NR1I2	1	1	2
VCAM1	1	1	2
IL-6	0.66666667	0.75	2
CYP1A1	1	1	2
CYP1A2	1	1	2
CYP3A4	1	1	2
HMOX1	0.66666667	0.75	2
IGF2	0.66666667	0.75	2
STAT1	0.83333333	0.8	3
MMP1	1	1	2
AHSA1	1	1	2
TNFSF15	1	1	2
BAX	1	1	2
JUN	1	1	3
SLC6A4	1	1	2
SLC6A3	1	1	2
ADRA1A	1	1	2
CHRM4	1	1	2
CRP	0.66666667	0.75	2
KCNH2	1	1	2
ADRA1D	1	1	3
ADRB2	1	1	6
ADRA2C	0.66666667	0.75	2
HTR3A	1	1	2
ADRB1	1	1	2
NCF1	1	1	2
RELA	1	1	3
MAOB	1	1	3
ACHE	1	1	6
F7	1	1	3
NCOA1	1	1	2
CCNA2	1	1	4
GSK3B	0.85714286	0.77777778	5
MAPK14	1	1	4
PPARG	1	1	10
ESR1	1	1	6
SLC6A2	1	1	2
RXRA	1	1	8
GRIA2	1	1	2
GABRA1	1	1	7
ADRA1B	0.85714286	0.77777778	5
CHRM2	0.7	0.71428571	3
AKR1B1	0.7	0.71428571	3
CHRM1	1	1	6
CHRM3	1	1	4
NCOA2	1	1	10
PRSS1	0.95454545	0.85714286	10
CHEK1	1	1	5
ESR2	1	1	5
PTGS2	1	1	13
SCN5A	1	1	6
AR	1	1	7
PTGS1	1	1	11
NOS2	0.97222222	0.9	8
PGR	1	1	4

**Table 4:** The docking information of 10 targets with the corresponding compounds (AC: active component; HG: hub gene; and TG: total score).

TS AC HG	Quercetin	Kaempferol	Formononetin	Isorhamnetin	Calycosin	7-O-Methylisomucronulatol
AKT1	4.384	4.880	4.527	4.94	4.831	5.579
VEGFA	5.491	5.637	4.655	5.958	5.844	4.948
IL-6	4.872	3.905	3.930	5.079	4.510	6.356
PPARG	4.156	5.435	5.499	5.368	5.201	6.072
CCL2	4.534	4.078	3.547	3.678	3.240	4.068
NOS3	6.667	5.571	4.824	5.804	6.195	6.632
SERPINE1	5.663	7.337	5.178	5.376	7.084	6.344
CRP	4.976	4.401	2.694	4.193	3.107	2.873
ICAM1	5.564	5.402	6.022	5.678	6.972	5.181
SLC2A4	5.233	6.075	5.318	5.196	6.399	5.761
	(3S,8S,9S,10R,13R,14S,17R)-10,13-Dimethyl-17-[(2R,5S)-5-propan-2-yloctan-2-yl]-2,3,4,7,8,9,11,12,14,15,16,17-dodecahydro-1h-cyclopenta[a]phenanthren-3-ol	Mairin	Jaranol	Hederagenin	3,9-di-O-Methylnissolin	9,10-Dimethoxypterocarpan-3-O-*β*-D-glucoside
AKT1	4.054	2.436	4.086	3.927	4.048	5.957
VEGFA	5.664	3.981	4.960	5.764	4.126	6.494
IL-6	7.126	4.740	5.825	6.287	3.916	6.114
PPARG	6.152	3.791	5.732	6.064	6.389	7.351
CCL2	3.36	3.132	3.921	4.741	4.247	6.563
NOS3	7.225	-0.347	5.974	8.568	7.354	6.169
SERPINE1	1.407	-14.035	6.437	2.694	5.141	7.152
CRP	4.919	1.003	3.218	4.336	3.006	4.061
ICAM1	8.581	2.007	5.178	5.339	5.538	6.059
SLC2A4	8.644	5.528	5.686	9.449	6.242	7.901
	(6aR,11aR)-9,10-Dimethoxy-6a,11a-dihydro-6h-benzofurano[3,2-c]chromen-3-ol	Bifendate	FA	1,7-Dihydroxy-3,9-dimethoxy pterocarpene		
AKT1	4.012	7.164	5.873	4.508		
VEGFA	3.602	5.177	8.093	7.426		
IL-6	3.976	4.045	6.541	5.065		
PPARG	5.152	6.364	7.445	4.521		
CCL2	4.278	5.238	5.059	4.052		
NOS3	6.520	3.976	9.906	5.907		
SERPINE1	5.027	2.737	8.245	5.963		
CRP	3.567	2.891	5.173	2.986		
ICAM1	5.227	6.795	9.754	5.231		
SLC2A4	4.837	7.733	8.877	5.041		

## Data Availability

The datasets used or analyzed during the current study are available from the corresponding author on reasonable request.

## Abbreviations

DN: Diabetic nephropathy
GO: Gene ontology
KEGG: Kyoto Encyclopedia of Genes and Genomes
PPI: Protein-protein interaction
ESRD: End-stage renal disease
DM: Diabetes mellitus
RAAS: Renin–Angiotensin–Aldosterone System
TCM: Traditional Chinese medicine
ACEI: Angiotensin-converting enzyme inhibitor
ARB: Angiotensin receptor blocker
TCMSP: Traditional Chinese Medicine Systems Pharmacology Database and Analysis Platform
OB: Oral bioavailability
Caco-2 cells: Intestinal epithelial permeability
DL: Drug-likeness
BBB: Blood-brain barrier
HL: Drug half-life
LR: Lipinski's rule
OMIM: Online Mendelian Inheritance in Man
AKT1: RAC-alpha serine/threonine-protein kinase
VEGFA: Vascular endothelial growth factor A
IL-6: Interleukin-6
PPARG: Peroxisome proliferator-activated receptor gamma
CCL2: C-C motif chemokine 2
NOS3: Nitric oxide synthase, endothelial
SERPINE1: Plasminogen activator inhibitor
CRP: C-reactive protein
ICAM1: Intercellular adhesion molecule 1
SLC2A4: Solute carrier family 2, facilitated glucose transporter member 4
GOBP: Gene Ontology biological process
GOCC: Gene Ontology cellular component
GOMF: Gene Ontology molecular function
PPAR*γ*: Peroxisome proliferator-activated receptor-*γ*
GMCs: Glomerular mesangial cells
PKB: Protein kinase B
ARB: Angiotensin type 1 receptor blocker
PlGF: Placental growth factor
ECM: Extracellular matrix
NO: Nitric oxide
DIGD network: Drug-ingredients-gene-disease network
AC: Active component
HG: Hub gene
TG: Total score.
